# New Dental Institute in Berlin

**Published:** 1885-02

**Authors:** 


### New Dental Institute in Berlin.

A new dental institute, established by the exertions of the Minister of Education, Herr Von Gorsler, was opened on October 15th. Professor Dr. F. Busch has been appointed director, and he will be assisted by Messrs. Paetsel, Muller, and Sauer, as instructors in theoretical and practical dentistry. The want of such a central institute has been felt. Hitherto, a large number of German students in dentistry have been obliged to seek their instruction abroad. The lectures for the ensuing term are on the following subjects: 1. Practical Surgery. 2. Diseases of the Teeth and Mouth. 3. Theory of Tooth-stopping. 4. Normal and Pathological History of the Teeth. 5. Theoretical Introduction to Mechanical Dentistry. The following practical courses are announced: 1. Policlinics of Diseases of the Teeth and Mouth, including the extraction of teeth. 2. Operative Dentistry, chiefly stopping decayed teeth. 3. Mechanical Dentistry, the supply of false teeth. For the general training of dental students in anatomy, physiology, physics, chemistry, and in the branches of practical medicine, there is plenty of opportunity by attending the lectures of the professors at the university.

